# Diversity, Biodiversity, Conservation, and Sustainability

**DOI:** 10.1100/tsw.2001.101

**Published:** 2001-10-11

**Authors:** Joao Carlos Marques

**Affiliations:** Institute of Marine Research, Department of Zoology, Faculty of Sciences and Technology, University of Coimbra, 3004-517 Coimbra, Portugal

**Keywords:** diversity, biodiversity, conservation, sustainability, indicators

## Abstract

The concepts of diversity and biodiversity are analysed regarding their historical emergence, and their intrinsic meaning and differences are discussed. Through a brief synopsis, difficulties usually experienced by statisticians in capturing the dynamics of diversity are analysed and main problems identified. The shift from diversity to the more holistic biodiversity as a working concept is appraised in terms of the novelty involved. Through a number of examples, the way the two concepts capture natural cyclic changes is analysed, and their reciprocal and complementary relations are approached theoretically. The way diversity could develop from the stores of biodiversity as its active expression through selective and evolutionary processes is described. Through the use of a very simple dynamic model, the concepts of diversity and biodiversity are analysed in extremely opposite hypothetical scenarios. Comparisons with natural situations are made and the theoretical implications from the conservation point of view are discussed. These support the opinion that conservation undertaken in restricted and protected areas is not self-sustainable, needing permanent external intervention to regulate internal processes, and in the long run will most probably lead in the direction of obsolescence and extinction. Finally, the relations between diversity, biodiversity, and sustainability are approached. The vagueness of the sustainability concept is discussed. Preservation of biodiversity is then defended as one of the best available indicators to assist us in fixing boundaries which may help to provide a more precise definition of sustainability.

### IS THERE A MEANINGFUL DIFFERENCE BETWEEN DIVERSITY AND BIODIVERSITY?

Biological diversity is a widely accepted concept which is usually defined as biological variety in nature. This variety can be perceived in an intuitive way. On the other hand, it has been assumed that it can be quantified and adequately expressed in any appropriated manner. Biological diversity has appeared as an interesting concept since we noticed the regular way individuals fall into species, which could be observed, for instance, through the study of insect collections, marine invertebrate samples, or bird counting during surveys. In randomly collected samples, besides the variation in the total number of species, it is easy to verify that a few species are always represented by many individuals, while decreasing numbers of specimens usually represent the remaining ones, and that many species are often represented by single individuals (Fig. 1). A tentative generalisation of such representation easily justifies describing a rocky shore community as more diverse than a sandy beach one. Needless to say, in practice, the approach will be very much dependent on the taxonomic characteristics of the group on which diversity estimations are tentatively based. In fact, as an example, it is clear that bacteria or algae present totally different problems than marine benthic invertebrates, zooplankton, or fish.

Of course, over time the scientific view of what the concept involves has changed, and nowadays diversity may be better regarded as the amount of genetic non-redundant information existent in a defined ecological (biological) system. From a quantitative point of view, the diversity of a given genetic pool (G) may be roughly expressed as a function of the number of species (S), of the biomass (B), or of the total number of individuals (N). Nevertheless, ecologists know well that we should expect more constancy in ratios than in absolute numbers. Moreover, ecologists are also familiar with the properties of logarithmic relations (e.g., allometric). Taking

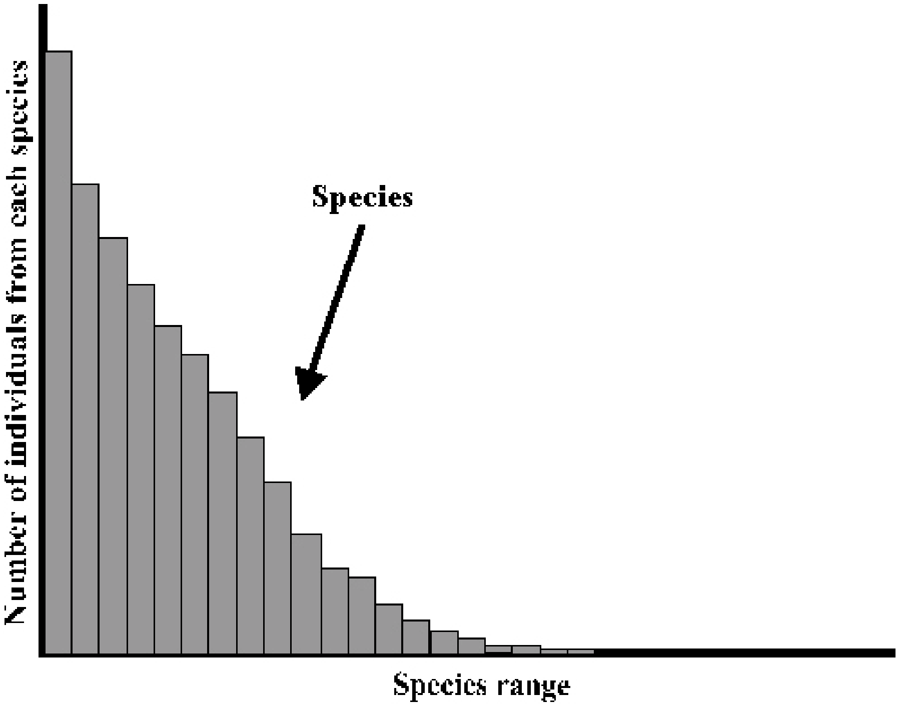

**FIGURE 1.** In a random sample, species can usually be ranged as a function of the number of individuals representing them.

these aspects into account, diversity could be tentatively expressed assuming that $G \approx S^k \approx B^{k'} \approx N^{k''}$.

Certainly, other more complex mathematical algorithms, have been used to express the same kind of relations. For instance, Volterra[1] proposed a satisfactory approach to what is basically the same problem and Fisher et al.[2] examined to what extent it was adequate to use expressions based on statistical hypothesis. We may nevertheless say that, in general, statisticians were never at ease with the diversity concept. The main reason for this might be the dynamics of diversity, which are permanently changing the information content, representing a difficulty in any set of definable probabilities. In fact, it is basically impossible to stabilise variance in samples, since the entire model suffers from the typically irregular dynamic change almost always found in nature. Such dynamics combine a general tendency to increase diversity through different processes with the occurrence of unexpected periods of decline, which are often spatially extensive. Therefore, although diversity can be measured, it can hardly be considered as a static property of samples proceeding from a given system.

The panoply of diversity indices is immense, and it is not the objective of this paper to repeat a critical review of it. Nevertheless, a few relations regarding the composition of natural communities exhibit relatively low spatial (local) and temporal variability, and therefore constitute suitable possibilities to be used as diversity measures. That is the case, for instance, of measures of information or of “entropy”, like the Shannon-Wienner index[3]:

$$H' = - \sum_{i=1}^{n} p_i \, Log_2 \, p_i \qquad (1)$$

where n = the number of species, and $p_i$ = the proportion of the number of individuals or biomass of species i in a community where the species proportions are $p_1$, $p_2$, $p_3$, ...$p_i$, ...$p_n$.

The Shannon-Wienner index is very much equivalent to the Brillouin index[4]:

$$H = (1/N) \, Log2 \, (N!/N_1! \, N_2! \, N_i!) \qquad (2)$$

It is easily demonstrable that diversity, when expressed by the Brillouin’s algorithm, will increase by $(1/N) \, Log2 \, [N_a/(N_b+1)]$ if, in a sample, one individual of a common species (a) is removed, being replaced by one individual of a more rare species (b). This is not too different from Volterra’s expression[1,5], which introduces a function of the sum $\sum a_i N_i \log N_i$.

It has often been tried (see for instance the two indices above) to discriminate between the two components of diversity, i.e., the number of species in presence (species richness) and the uneven representation of the different species (evenness). Nevertheless, the two components are correlated, since they emerge from an artificial division of the same distribution pattern, produced by our minds, and therefore it can be argued that there is no reason why we should expect any clear advantage from such an approach.

Another experiment is represented by the “rarefaction methods,” or extrapolation procedures, which seek to estimate an “expected number of species.” Nevertheless, the statistical properties in the natural sets assumed by such methods, although interesting, are not proven, and therefore cannot be recommended.

Even one of the simplest ways to express diversity, the rank-frequency diagrams[6], may be criticised, when certain features of the sequences, like “convexities” or “concavities,” are interpreted as an effect of prevailing biological interactions. Although this might be possible, the most probable, and perhaps more realistic, explanation for such characteristics is that the initial conditions of the average pattern may simply reflect transient population oscillations affecting the species living concurrently in a given community. On the other hand, this constitutes an interesting starting point regarding the issue of seasonal variation and its irreversibility.

An additional problem in estimating diversity must be examined. It lies in the fact that modular organisms (e.g., large marine macrophytes or macroalgae) cause difficulties in defining and counting individual organisms as such. Consequently, it becomes much more complicated to estimate diversity when we deal with systems that combine big vegetal species or colonial animals with isolated individuals often belonging to a great number of different size strata. In such cases, to perform estimations based on biomass provides only a partial solution. In fact, modular species are of great importance, namely because they behave as quite conservative genetic pools. In other words, nature is too complex to be successfully described by simple indices, and all the attempts to propose new ways to estimate diversity could not provide any tangible conceptual progress[3].

This brief synopsis provides an approximate idea of the difficulties involved in understanding the dynamics of diversity. Therefore, it is not pessimistic to say that there is no conceivable "diversity index" capable of expressing the dynamics of mixed populations, exhibiting stabilised values through space and time. The difficulties may be summarised as follows:

1. The increase of diversity through time is inevitably gradual, more often than not associated with the emergence and transformation of an organised system, but the decrease of diversity is most frequently abrupt.
2. Looking to the spatial characteristics of ecosystems, we are forced to conclude that it is impossible to have stabilised variance, which may lead us to favour any kind of spectral expression taking into account the way diversity may shift as a function of the space considered. The problem in this case is that each spatial enlargement provides a different spectrum as a function of the characteristics of new sites added to the sample.
3. Since the biosphere is continuous, it is not adequate to set apart "local" diversity (called $\alpha$ diversity) from diversity estimated by pooling discontinuous patches ($\beta$ diversity) or measured at larger spatial scales ($\gamma$ diversity), although, to a certain extent, such descriptions might become helpful to approach the biodiversity concept.

We may say that, at present, biologists give less emphasis to the interpretation of diversity as an indicator of representative segments of the biosphere. Instead, their interest has turned to "biodiversity." This new term emerged suddenly at the end of the 1980s, and its use expanded rapidly. Although it is still difficult to say where this new concept came from, biodiversity is, beyond any doubt, much more "saleable" and therefore much more appealing to decision makers. However, we need to appraise the term to determine if its novelty restricts it to a "marketing" concept.

To some authors, biodiversity may be seen as the full range of biological diversity, from intraspecific genetic variation to the species richness, connectivity, and spatial arrangement of entire ecosystems at a landscape level scale (see, for instance, Solbrig[7]). This means that biodiversity is much less concerned with the existing numerical proportions among different taxonomic units and much more with the absolute number of such taxonomic units or, in other words, genetic richness. In this sense, biodiversity represents a more holistic approach to an ecosystem's properties than the old diversity concept.

The biodiversity concept becomes especially interesting with regard to high diversity ecosystems, where the two properties, in their ultimate form, are not necessarily related to higher biological or metabolic activities. In fact, the opposite is often true. A good example of this is the profuse biological diversity in deep sea benthic environments, where the major driving force behind diversity development is the balance between the energy flow, usually expressed in turnover, and the genetic differentiation related to the difficulties of survival in such an environment.

Although more holistic, the biodiversity concept is indeed relatively simple, which has allowed it to relieve the pressure concerning quantitative measures that prevailed for many years following the more conventional concept of diversity. On the other hand, such a different perspective emphasised the goal of finding simple and comprehensive regularities that may relate the total number of species found in samples with environmental changes at a smaller (local) scale through speciation, migration, and extinction processes. The previous diversity concept gave rise to biodiversity, which finally helped in making its meaning more precise. In fact, through years of use, the old concept of diversity had been "side tracked" to a kind of static statistical problem, which, in fact, it is not since it expresses the dynamics of ecosystems as a whole. On the other hand, the emphasis on biodiversity allowed revitalising taxonomic studies (this was most probably one of the ideas behind the shift in concepts) and provides a better tool to assist in the analysis of the structure and dynamics of the biosphere. The relations between the two concepts are therefore significant and will be further analysed.

### Diversity, Biodiversity, and Natural Cyclic Changes

In ecosystems, diversity undergoes a normal cyclic change, i.e., in seasonally fluctuating climates. Such changes may be described through a series of irreversible loops on a system of coordinates relating, for instance, to the number of species (S) and the number of individuals (N), or the number of species and biomass (B). Let us use as an example the Mondego estuary benthic communities[8,9,10] located in a temperate climate. During the spring, the number of individuals and biomass both tend to increase, but at the very beginning of the season only a small number of species are present; at least only a few of them are able to start reproducing quickly, taking advantage of the newly available resources. The result is that diversity is low, independent of the measure we use. As summer moves onwards, diversity rapidly increases. Later, during the fall, many species still persist in the system, although presenting rather low densities. Finally, during the winter, when many species are dormant, diversity decreases significantly. Nevertheless, when we compute diversity, we are usually not taking into account eggs, algae spores, resting stages found in the mud, etc., as we should. Many other examples illustrating this pattern of variation in temperate ecosystems can be found in the literature.

The study of the annual cycle of diversity in ecosystems from the temperate zone is very helpful in understanding the relations and differences between the concepts of diversity and biodiversity. It appears reasonable to consider that diversity comes closer to biodiversity during the summer. In fact, diversity is usually an expression of the representation of species in the active ecosystem, while biodiversity should be considered rather closer to the summer situation of any clearly seasonal ecosystem. Let us take another example: a temperate forest. In this case, seeds in the soil of the forest surely contains many potentialities that do not have any expression in diversity measures based on winter samples. The same goes for the aquatic species, or their resting stages, present in riverbanks, where they wait for the proper occasion to recolonise the benthic communities. In other words, they constitute a reserve of biodiversity that may show up when environmental conditions allow ecological succession to continue.

### Diversity Develops from the Stores of Biodiversity

Another example may better illustrate the relations between the concepts of diversity and biodiversity as they are generally used. It is well known that, independent of local diversity in marine plankton communities, plankton blooms associated with upwelling, which usually develop successively or in different places, concern only a few species. This is in many aspects similar to what we described with regard to cyclic changes in temperate terrestrial and aquatic ecosystems. Since in biodiversity we comprehend the reserve of genotypes (see above), such marine plankton blooms may be interpreted as "experiments in evolution" which borrow genotypes from the reserve of biodiversity, play with it and, genetically changed or unchanged, return it back to the general store. The major interest of ecologists is typically focused on the most active segments of the yearly spectrum, associated with moderate or high biological activity, here referred to as "experiments in evolution." From this perspective, it might be suggested that in temperate regions we should usually find a significant difference between actual diversity and total potential (bio)diversity, while in equatorial climates, where seasonal variations are meaningless, the difference between diversity and biodiversity should not be so large[11].

To look upon biodiversity as a dynamic reserve containing a fraction of the results from past evolutionary processes, acting as a genetic information pool and providing the potential substratum for existent diversity, is quite helpful in enlightening the relations between the two concepts (Fig. 2). In accordance with this perspective, ecosystems should continuously accede to genetic materials from the biodiversity store, and natural selection should act upon their active expression. In case any new valuable genetic acquisition occurs, eventually new information provided by the reworked materials returns to the biodiversity store, enriching it. On the other hand, over time, the parts of the biodiversity store that have less and less actual active expression should become obsolescent and eventually disappear, together with much larger fractions of information that are removed as a result of major environmental changes, e.g., orogenic processes, climatic change, or human impacts. Events taking place in the active parts of ecosystems are forcibly constrained within limits that depend on the biodiversity store, and must be seen as "experiments in evolution" that provide a rather important loop, necessary to understand the reciprocal relations between the complementary concepts of diversity and biodiversity.

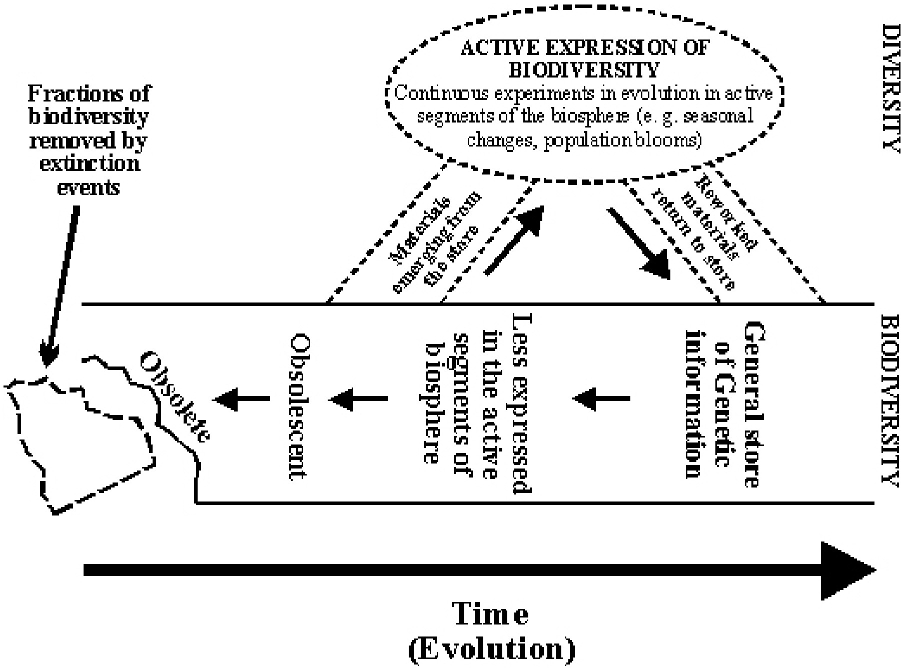

**FIGURE 2.** Tentative scheme describing the relations between diversity and biodiversity.

## RELATIONS WITH CONSERVATION AND SUSTAINABILITY

### Conservation Perspective

It might be interesting to analyse the concepts of diversity and biodiversity in extremely opposite situations through the use of a very simple dynamic model. Let us consider a scenario corresponding to an extreme situation: a flow culture submitted to a strong selection for yielding. The stock able to multiply fastest will become the lasting one, and the final situation will be a monospecific population with minimal diversity. Let S be the number of species, i.e., discernible genotypes, and N the number of individuals, or total biomass. The final scenario referred to above represents the situation where the turnover or flow are maximised and diversity is minimal, which can be described by

$$S = N^k \quad (3)$$

when $k \rightarrow 0$, we have a suitable indicator of minimal diversity.

In practice, what occurs in the flow culture is a selection of genotypes able to adhere to the walls, having as a result that a fraction of the individuals escapes the risk of being washed out from the system. Actually, this scenario resembles (to a certain extent) the selective and evolutionary processes that take place in rivers, where the capability of species to resist being washed away usually favours a lower investment in reproduction. Such an evolutionary pattern is common in rivers and, as a consequence of different possibilities open to slow down drift, benthic diversity is usually higher than plankton diversity.

Let us consider now an example of a diametrically opposed scenario. We can imagine an artificial system, i.e., a zoological garden, which goes on receiving new specimens but from which all the extra individuals from the species already represented are systematically removed. As a consequence, a situation of maximum diversity would be created. In such case

$$S = N^1 \quad (4)$$

and therefore the value of k, as proposed in Eq. 3, would be 1. The idea of proposing a scale of diversity between 0 (all specimens belonging to the same species) and 1 (all specimens belonging to different species) appears immediately as the next step, and k could be a potential diversity index.

These examples suggest that a system with maximal diversity (biodiversity) is somehow artificial and needs to be supported through processes that are controlled from the outside. We may, to a certain extent, extrapolate that this supports the opinion that conservation undertaken in restricted and protected areas, not to say in botanical and zoological gardens, is not self-sustainable, needing a permanent external intervention to regulate internal processes. In the long run, conservation carried out in such conditions will most probably lead in the direction of obsolescence and extinction. This point of view may indeed seem very pessimistic, but it is in agreement with observations in some natural ecosystems that present high diversity, although often exhibiting low turnover and low P/B ratios. Moreover, such systems almost always receive a significant supply of matter from external sources. This is exemplified by the dynamics and composition of deep-sea communities[12], which energetically benefit from the regular tumble of organic materials and nutrients proceeding from the upper levels.

Margalef[11,13] proposed a very simple model comparing two feedback loops. The first feedback concerned the total biomass or the total number of individuals:

$$dN/dt = aN^h - bN^{h'} \quad (5)$$

Margalef's model does not use the entire powers 1,2, as is done in the usual "demographic approach." Instead, it uses any values of h and h', provided that h' > h, which is necessary to have regulation. The second feedback loop concerned the changes in number of species, and was inspired in the hypothesis of island biogeography[14]:

$$dS/dt = cS^{m} - dS^{m'} \qquad (6)$$

Let us assume that a relative steady state may be approached, where $dS/dt \rightarrow 0$. In such case, the diversity index k (see above) becomes:

$$k = \log S/\log N \text{ or } k = B\,(h'-h) / (m'-m), \text{ after making } B = \log (bc/ad) \qquad (7)$$

Therefore, here k expresses the relative efficiency of the two feedback loops, which regulate respectively the total biomass or number of individuals and the total number of species. The differences between the powers, h'-h and m'-m, define how diversity will stabilise. Of course, we could propose more correct mathematical expressions to express the same relations, although that may be more complicated. Besides, it is not likely that the assumed constants are really so, which makes the whole exercise a waste of time. In point of fact, regarding an ecosystem's response to changing environmental conditions, the aim of this simple approach was merely to show that:

1. If it is easier for the system to increase biomass than to split the populations genetically speaking, then local diversity will decline.
2. If the biomass or number of individuals cannot increase, although genetic splitting can ensue or colonisation from outside is likely to occur, then local diversity will increase.

The old time-stability hypothesis[15], for instance, may be interpreted in conformity to Margalef's model[11]. According to Sanders[15], the characteristics of marine communities modify in space and time as a function of changing environmental stress. More precisely, at the local level, a long period of low environmental stress would make possible the development of what Sanders called "biologically accommodated" communities, while in more highly stressed environments we should expect to find "physically controlled communities," up to a level of stress beyond any possible adaptation of living organisms. Estuaries constitute a paradigm of "physically controlled communities," corresponding to the situation described in (1), while deep-sea and rocky-shore infralittoral communities are good examples of "biologically accommodated" communities, corresponding to the situation described in (2).

Likewise, the same model can be applied to other situations. Natural succession change, for instance, tends to evolve from a type (1) to a type (2) situation in a rapid way, and biogeographically speaking, high to low latitude ecosystems also change according to the same pattern[11].

### Sustainability Perspective

Sustainability and sustainable development are two recent concepts that entered more and more in our terminology in the last decade, although their meaning remains somehow imprecise, namely when it comes to application. From a pessimistic point of view, if development should imply an indefinite increase of human population and/or in the consumption of energy, there will be obviously no way to sustain it, although we may decide to rely upon any future extraordinary scientific/technological solution. Since such solution is not yet known in any case, we may also assume optimistically that an increase in quality of life, as we understand it now, does not necessarily imply a substantial increase of energy consumption, and therefore that there is such a thing as the possibility of sustainable development.

There is no clear definition of what a sustainability niche might be, but as a rough approach it is commonly accepted that the present human population should use natural resources in a way that these remain a patrimony for the future generations. Under the risk of an irreversible destruction of most of our actual renewable resources, therefore compromising the future, this will imply that we must preserve the integrity of natural ecosystems.

Let us consider some of the definitions of ecosystem integrity found in the literature, although trying to define the concept might be, to a certain extent, contradictory to the meaning of the concept itself:

- “Biological integrity is the maintenance of the community structure and function characteristic of a particular local or deemed satisfactory society” (Cairns, in Kay[16]);
- “Biological integrity is the capability of supporting and maintaining a balanced, integrated, adaptive community of organisms having a species composition and functional organisation comparable to that of the natural habitat of the region” (Karr and Dudley, in Kay[16]);
- “If a system is able to maintain its organisation in the face of changing environmental conditions then it is said to have integrity” (Kay[16]).

These different definitions, formulated over more than a decade, evolved from a more motionless to a more dynamic notion of integrity, but the system’s capabilities to maintain organisation and function remain the central concept. A decrease in available organic information, as we should expect to occur in association with a loss of biodiversity, may impair somehow the capacity for obtaining an optimised rate of change in any future recovery from disasters caused by the pressures of human population. It is surely not possible to prevent a fraction of the organic information available in the biosphere becoming obsolescent, and finally be lost, as a consequence of changes introduced by humankind. In accordance with the approach presented in this paper, we should expect human pressure to cause first a decrease in diversity and, after a short delay, in biodiversity as well. To this effect we must add the result of disturbances of a more fortuitous and catastrophic character, natural or human made, taking away large fractions of biodiversity. In the short term, this will endanger the capacity of present ecosystems to maintain organisation and function, that is to say, their integrity. And the loss of ecosystem integrity definitely does not fit in the concept of sustainable development. Therefore, the preservation of biodiversity is perhaps one of the best indicators we may use to fix boundaries that help us define, in a more precise way, the meaning of this concept.

### CONCLUSIONS

Diversity and biodiversity indeed constitute different concepts. A suitable description of the relations between them can be visualised if we consider diversity, which is actually what we can measure directly, as developing from stores of biodiversity.

Taking diversity and biodiversity as ecological indicators, it seems reasonable to assume that conservation undertaken in restricted and protected areas is not self-sustainable, needing permanent external intervention to regulate internal processes.

Despite difficulties in providing an operational definition of sustainability, the preservation of biodiversity appears to be one of the best indicators to assist in decision making with regard to sustainable development.
